# 肺淋巴管平滑肌瘤病的临床病理分析

**DOI:** 10.3779/j.issn.1009-3419.2011.04.14

**Published:** 2011-04-20

**Authors:** 俊 高, 培菊 朱, 尚福 张, 莎 赵, 昌立 鲁, 卉娇 陈

**Affiliations:** 610041 成都，四川大学华西医院病理科 Department of Pathology of West China Hospital, Sichuan University, Chengdu 610041, China

**Keywords:** 肺肿瘤, 淋巴管平滑肌瘤病, 免疫组化, 诊断, Lung neoplasms, Lymphangioleiomyomatosis, Immunohistochemistry, Diagnosis

## Abstract

**背景与目的:**

肺淋巴管平滑肌瘤病（pulmonary lymphangioleiomyomatosis, PLAM）是一种罕见的具有独特临床病理特征的肿瘤性疾病，本文旨在探讨PLAM的临床病理学特点及其鉴别诊断。

**方法:**

收集3例PLAM病例，复习患者的临床资料，进行组织学及免疫组化观察，并结合文献复习分析。

**结果:**

3例PLAM病例均为育龄妇女，年龄27岁-45岁，平均年龄37.7岁，其中2例胸部CT示双肺弥漫分布薄壁的含气囊腔，1例为气胸。组织学检测显示肿瘤组织呈不规则腔隙和斑片或结节状，由内皮细胞及其周围大量增生的平滑肌样梭形细胞构成，可沿支气管、血管等分布。免疫组织化学检测示增生平滑肌样梭形细胞呈现结蛋白（Desmin, Des）、钙结合蛋白（Caldesmon, Caldes）、平滑肌肌动蛋白（α-Smooth muscle actin, SMA）、肌特异性肌动蛋白（Muscle-specifc actin, MSA）、人类黑色素瘤单克隆抗体（HMB-45）、黑色素瘤标记物（Melanoma Marker, CD63）、波形蛋白（Vimentin, Vim）、雌激素（Estrogen, ER）和孕激素（Progestogen, PR）阳性表达，而T细胞1识别的黑色素抗原（Melanoma Antigen Recongnized by T cell 1, MRT-1）为阴性表达，毛细血管内皮细胞第8因子（FVⅢ）、人类造血干细胞表面分子（CD34）阳性表达，淋巴管内皮细胞呈淋巴管内皮细胞标记物（D2-40）阳性表达。本组病例随访时间为3个月-25个月，生存状况良好，未见该病复发，除肺外其余各系统未见淋巴管平滑肌瘤病等。

**结论:**

PLAM的主要病理改变为沿支气管、血管分布的淋巴管周围平滑肌细胞异常增生，一般发病于绝经期前育龄女性，渐进性呼吸衰竭导致该病预后不良。

## Abstract (en)

**Background and objective:**

Pulmonary lymphangioleiomyomatosis (PLAM) is a rare tumor with unique clinicopathological features. The aim of this study is to investigate the clinicopathological features, the diagnosis and differential diagnosis of pulmonary lymphangioleiomyomatosis.

**Methods:**

Tree cases of PLAM were analyzed by light microscopy, immunohistochemistry and their clinical data, and the relative literatures were reviewed.

**Results:**

Tree cases of patients suffered from PLAM were the women in their reproductive aged, from 27 years to 45 years (mean 37.7 years), two cases of the HRCT showed bilateral diffuse cystic airspaces changed, and one case was the pneumothorax. The histopathological examination revealed the tumor was composed of the variably sized cystic spaces are lined by plaque-like or nodular aggregates of endothelial cells and the hyperplasia, smooth-muscle-like spindle cells which was along with the bronchi and the vessels. The immunohistochemistry showed that Des, Caldes, SMA, MSA, HMB-45, CD63, Vim, ER and PR were positive in the hyperplasia spindle cells, and there was no expression of MRT-1. The FVⅢ, CD34 were positive in the capillary endothelial cells, and the D2-40 was positive in the lymphatic vessels. All the patients were alive without the recrudescence of the PLAM since the diagnosis, about 3 months to 25 months, and there was no LAM in their other systems.

**Conclusion:**

The most signifcant histopathological feature of pulmonary lymphangioleiomyomatosis was the progressive invasion of smooth muscle cells into the lymphatic vessels, and the blood vessels. The majority of the cases occur in the lungs of the women in the predominantly premenopausal and middle-age. It is a poor prognosis due to the progressive respiratory failure.

肺淋巴管平滑肌瘤病（pulmonary lymphangioleiomyomatosis, PLAM）是原发于肺的一种罕见的肿瘤性疾病，可弥漫累及双肺。一般发生于育龄期妇女，常因自发性气胸、乳糜性胸水等导致呼吸功能不全。本文共收集3例PLAM，并结合文献对该病的临床病理特征及鉴别诊断等进行分析。

## 1 材料与方法

### 1.1 材料

在四川大学华西医院病理科1960年-2010年进行活体组织检查的213, 647例肺肿瘤中仅发现3例PLAM。

### 1.2 方法

3例PLAM标本均用4%甲醛固定，包埋，行HE染色，选用Des、Caldes、SMA、MSA、HMB-45、CD63、MRT-1、Vim、ER、PR、FVⅢ、D2-40和CD34等标志行免疫组化染色，免疫组化采用SP法，操作按试剂说明书进行，抗体及相应试剂均购自福州迈新生物技术开发有限公司。

## 2 结果

### 2.1 临床资料

3例PLAM均为育龄期妇女，年龄27岁-45岁，平均年龄37.7岁，均无相关疾病家族史。其中2例以咳嗽、气促等呼吸系统症状为主要临床表现。CT检查示双肺弥漫分布薄壁的含气囊腔（图 1）。1例临床表现为反复发作的自发性气胸，CT示气胸。3例患者的肺功能均受损。本组病例随访时间较短为3个月-25个月，均存活，随访期间未见该病复发，除肺外其余各系统均未见LAM等。

**1:**
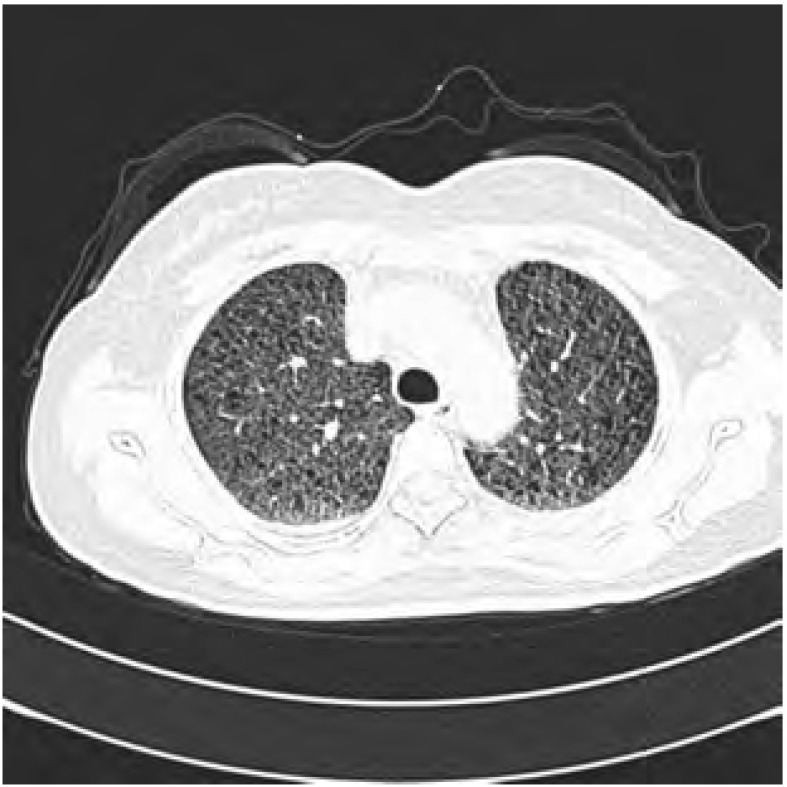
肺HRCT示双肺纹理增多，广泛分布大小不一的含气囊腔 High-resolution CT shows numerous small and sizable cysts distributed diffusely in both lung

### 2.2 病理检查

#### 2.2.1 大体标本观察

3例标本均为局部切除，表面光滑，略呈斑片或细颗粒的结节状，切面含大小不等的囊腔，其中1例表面呈肺大疱状。

#### 2.2.2 组织形态学观察

肿瘤组织由大小不等的囊肿和平滑肌样梭形细胞以及内皮细胞组成的斑片或结节状病变为主的两种病变构成，即不规则腔隙状的淋巴管及其壁上增生的平滑肌样梭形细胞组成。增生的平滑肌样梭形细胞形成小结节，呈漩涡状，并突入淋巴管腔内，支气管、血管周围的淋巴管均可见有增生的平滑肌样梭形细胞。增生的平滑肌样梭形细胞呈圆形或短梭形，胞质丰富，嗜酸性，核偏大，未见核分裂，周围可见含铁血黄素的巨噬细胞（图 2）。

**2:**
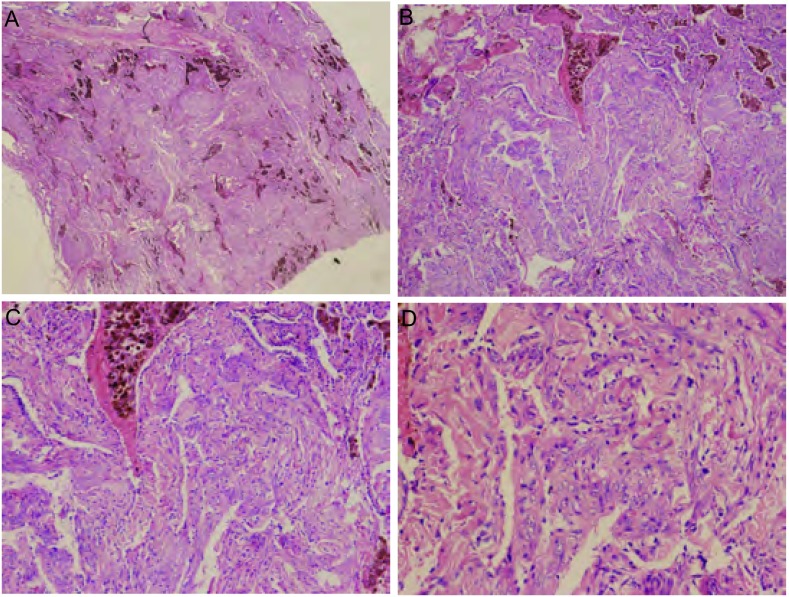
组织病理学观察。A：肿瘤组织呈不规则腔隙和斑片或结节状（HE，×5）；B：肿瘤组织沿管腔生长，可见含铁血黄素沉着（HE，×25）；C：肿瘤组织由呈不规则腔隙的淋巴管及其壁上增生的梭形平滑肌组织组成，沿支气管、血管束等分布（HE，×50）；D：肿瘤细胞呈梭形，胞质丰富，嗜酸性，核大，核仁明显，未见核分裂（HE，×100）。 Histopathology. A: The tumor grown along with the tubes shaped plaque-like, and hemosiderin deposited (HE, ×5); B: The tumor grown along with the tubes shaped burble, and hemosiderin deposited (HE, ×25); C: The tumor was composed of lymphatic vessels with abnormal lacunae and the spindle smooth muscle in the wall of the tubes, which was along with the bronchi and the vessels (HE, ×50); D: The spindle tumor cells contained abundant eosinophilic cytoplasm without mitotic figures arranged in broad (HE, ×100).

### 2.3 免疫组织化学检测

增生的平滑肌样梭形细胞呈Des、Caldes、SMA、MSA、HMB45、Vim和CD63等阳性表达，MRAT-1阴性表达。部分肿瘤细胞细胞核呈ER、PR阳性表达。增生的淋巴管内皮细胞呈FVⅢ、D2-40和CD34阳性表达（图 3）。

**3:**
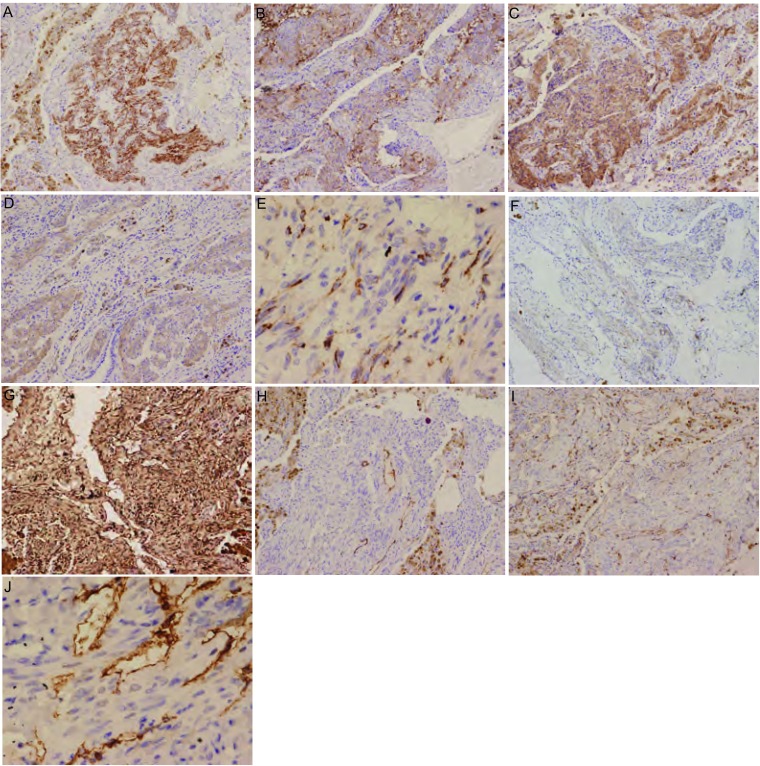
免疫组织化学染色。A：Des示肿瘤细胞的胞质呈阳性表达（SP，×50）；B：Caldes示肿瘤细胞的胞质呈阳性表达（SP，×50）；C：SMA示肿瘤细胞的胞质呈阳性表达（SP，×50）；D：MSA示肿瘤细胞的胞质呈阳性表达（SP，×50）；E：HMB-45示部分肿瘤细胞的胞质呈阳性表达（SP，×200）；F：CD63示部分肿瘤细胞的胞质呈阳性表达（SP，×50）；G：Vim示肿瘤细胞呈弥漫阳性表达（SP，×50）；H：FⅧ示毛细血管内皮细胞胞质呈阳性表达（SP，×50）；I：CD34示毛细血管内皮细胞胞质呈阳性表达（SP，×50）；J：D2-40蛋白示淋巴管内皮细胞胞质阳性表达（SP，× 200）。 Immunohistochemistry. A: Des was positive in almost cytoplasm of all tumor cells (SP, ×50); B: Caldes was positive in almost cytoplasm of all tumor cells (SP, ×50); C: SMA was positive in almost cytoplasm of all tumor cells (SP, ×50); D: MSA was positive in almost cytoplasm of all tumor cells (SP, ×50); E: HMB-45 was positive in almost cytoplasm of some tumor cells (SP, ×200); F: CD63 was positive in almost cytoplasm of some tumor cells (SP, ×50); G: Vim was strong expression in all tumor cells (SP, ×50); H: FⅧ was positive in cytoplasm of capillary endothelial cells (SP, ×50); I: CD34 was positive in cytoplasm of capillary endothelial cells (SP, ×50); J: D2-40 was positive in cytoplasm of endothelial cells of lymphatic vessel (SP, ×200).

### 2.4 病理诊断

本组3例患者在发病年龄、临床资料及病理检查上，均为较典型的PLAM。

## 3 讨论

PLAM是一种罕见的肿瘤性疾病。Von Stossel等与Burrel等分别于1937年首先报道该病。1966年Cornog等^[1]^将该病命名为“肺淋巴管平滑肌瘤病（PLAM）”。1993年龚俊辉等^[2]^报道了我国首例PLAM。WHO软组织和骨肿瘤分类（2002年）已经将此病归入软组织的血管周上皮样细胞分化的肿瘤（neoplasms with perivascular epithelioid cell differentiation, PEComas）中，即一组组织学和免疫表型上具有血管周上皮样细胞特征的间叶性肿瘤^[3]^。该病罕见，我国尚无该病确切的流行病学资料，国外有关此病的患病率报道也不一致。2010年1月《欧洲呼吸杂志》发表的欧洲呼吸协会制定的PLAM新指南认为PLAM分为特发性和继发性，成年女性特发性的PLAM发病率约为1/400, 000。在复习四川大学华西医院1960年-2010年进行活体组织检查的肺肿瘤213, 647例中，仅发现PLAM 3例。该病绝大多数发生于育龄妇女，70%在20岁-40岁发病，平均发病年龄为（32±8.9）岁。但文献报告中也有个别青春期前和少数绝经期女性发病。个别文献^[4, 5]^报道结节性硬化症的男性也可同时继发PLAM。

目前对PLAM的病因、发病机制尚未十分清楚。PLAM既可以作为结节性硬化病（tuberous sclerosis complex, TSC）累及肺部的表现，亦可作为一种散发的、非遗传性疾病^[6]^。有报道PLAM患者同时患有血管平滑肌脂肪瘤（angiomyolipoma）^[7]^。近年来有研究^[8]^提出，作为PEC瘤中的PLAM，其发生发展与基因*TSC1*（9q34）或*TSC2*（16p13.3）缺失相关，该基因的缺失可激活哺乳动物雷帕霉素靶蛋白（mammalian target of rapamycin, mTOR）通路，导致细胞增殖。虽然PLAM在病理组织形态上为良性病变，但几乎均为肺内广泛性的、多发性的肿瘤病变，故其发生发展极可能与某些基因异常有关。但也有学者^[9]^考虑PLAM具有明显的性别特征而认为雌激素在该病的发病中起重要作用。

活动后气促、咳嗽、咯血、乳糜胸及反复发作的自发性气胸为本病主要临床表现。本组病例中2例患者以咳嗽、活动后气促为主要症状，肺功能改变多数为阻塞性通气功能障碍及弥散障碍，也可表现为限制性或混合性通气功能障碍^[10-12]^。本病几乎均为肺内广泛性的、多发性的肿瘤病变，如果病变占据多数肺组织，将严重影响患者的肺功能，导致其功能障碍。早期X线胸片主要表现为双肺呈广泛网格状、结节状或粟粒状，晚期可表现为蜂窝状。高分辨率CT是本病最具有诊断价值的影像学检查，其典型改变为双肺纹理增多，交织成网状，可见薄壁囊腔弥漫分布，囊壁厚薄不均，厚度一般 < 1 mm，小囊腔可融合，囊腔直径可以从数毫米到数厘米，以2 mm-5 mm为主。有报道认为囊腔的大小和形态与本病的严重程度有关。部分患者伴有纵隔淋巴结肿大、气胸、胸腔积液、胸导管扩张等^[12]^。

PLAM的组织病理学改变为肉眼上早期病变类似肺气肿，较晚期表现为双肺囊腔，呈蜂窝状，若间质纤维化时可形成灰白色厚间隔，并伴有胸膜增厚。囊腔弥漫分布，直径从数毫米到数厘米，可含空气、血清样或乳糜样液体。若病变累及腋下、颈部、锁骨、纵隔等淋巴结，则呈苍白的海绵样改变；若累及胸导管则呈腊肠样改变^[12]^。镜下，肺组织中普遍增生的梭形平滑肌细胞，突入淋巴管，可累及肺泡壁、血管壁和支气管壁，呈结节漩涡状，使管壁增厚，可引起局部管腔阻塞。在病变早期，肺泡壁内集中大量增生梭形的平滑肌细胞，肺泡壁周围水肿、出血，可见含铁血黄素，形成结节。随着病变发展，结节变大伴束状胶原纤维增生，使肺泡壁破坏、囊腔形成，囊腔壁主要由平滑肌细胞、肺泡上皮细胞组成。若累及支气管、淋巴管，则管腔狭窄、堵塞；若累及肺小血管，可见动脉壁增厚、静脉栓塞等；若淋巴结受累则可见平滑肌生长包裹淋巴窦，呈交织状^[6, 12]^。电镜下可见淋巴管被覆扁平的内皮细胞，其下的基板不明显，有细丝将内皮细胞固定于胶原纤维中。紧邻胶原纤维有束状排列的平滑肌细胞；平滑肌细胞分化较成熟，呈梭形，有细胞核和丰富的肌丝，肌丝间有密体，质膜下有密斑，可见线粒体和内质网等少量细胞器；在毛细血管和肺泡上皮下均可见大量增生的梭形平滑肌细胞^[13]^。仅凭组织病理对PLAM的诊断较为困难，尤其是纤维支气管镜活检的小组织，故免疫组织化学染色十分重要。PLAM免疫表型检测中可见增生的平滑肌细胞SMA、Des、MSA、Caldes等肌源性抗体阳性表达和Vim阳性表达，部分平滑肌细胞PR、ER阳性。大部分PLAM细胞有许多特点与PEC瘤家族相似，例如HMB-45、MRT-1和CD63等阳性，尤其HMB-45阳性表达对诊断该病具有重要意义，正常肺组织或其它肺弥漫性病变时HMB-45均为阴性，同时淋巴管内皮细胞可有FVⅢ、CD34和D2-40等阳性表达。另据Martignoni等^[14]^报道，PLAM也可表达眼球相关转录因子（microphthalmia-associated transcription factor m, MiTF）。同时Chilosi等^[15]^使用免疫组织化学的方法研究前组织蛋白酶原K（cathepsin-k）在PLAM、肾血管平滑肌脂肪瘤、机化性肺炎、间质性肺炎和肺气肿组织中的表达情况发现，cathepsin-k在PLAM和肾血管平滑肌脂肪瘤中呈强阳性表达，并认为cathepsin-k有望在PLAM的病理诊断中起重要作用。

在本病的诊断过程中，需与TSC累及肺组织相鉴别。TSC累及肺部与PLAM的组织病理改变相同，不同的是TSC为常染色体显性遗传病，25%-50%患者有家族遗传史^[12]^，且该病多器官受累，包括脑、肺、视网膜、皮肤、肾脏及心脏等，故诊断PLAM时须排查多个系统以鉴别TSC。

PLAM还需与肺平滑肌瘤病（leiomyomatosis of the lung）鉴别诊断。后者大体病理改变与其它部位的平滑肌瘤病理改变类似，表现为单个或多个结节，切面呈漩涡状。组织形态表现为肺组织内单个或多个结节，呈膨胀性生长，结节由梭形肿瘤细胞构成，推挤周围组织；梭形细胞呈束状或编织状排列，形似正常的平滑肌细胞，胞质丰富，苏木素-伊红（HE）染色示细胞质红染，细胞核呈短梭形，未见核分裂，无出血、坏死等。免疫组织化学示肿瘤细胞具有平滑肌细胞的分化特征，即Desmin、α-SMA、MSA和Actin呈阳性表达，而HBM-45、FVⅢ、D2-40和CD34呈阴性表达。

迄今尚无对照研究来评价本病的最佳治疗方案。放疗及皮质类固醇激素治疗无效，对化疗也未有大量研究。目前治疗措施主要是抗雌激素治疗，包括孕激素治疗和卵巢切除术等，可减缓病程进展速度，但疗效不肯定^[6]^。肺移植可能是晚期患者最有效的治疗方法，但移植后可复发，目前认为可能与PLAM肿瘤细胞转移到移植肺有关。约50%的患者移植后可存活3年^[16]^。本病预后各不相同，自然病程平均8年-10年，发病后5年、10年、15年生存率分别为91%、79%、71%。近年文献^[17]^报告PLAM预后并没有改善，呼吸困难为主的患者预后差。Matsui等^[18]^提出PLAM患者的5年和10年生存率如该病的组织学评分（LAM histology score）为LHS-1是100%，LHS-2分别是89.9%和74.6%，LHS-3分别是59.1%和47.3%，并发现含铁血黄素沉着程度的增加与高的LHS的分数（*P*=0.029）有关，且预后差（*P*=0.001, 2）。本组病例随访时间较短，仅为3个月-25个月，本病的预后情况还需更多病例观察和更长时间随访。
